# Spatial and Structural Metrics for Living Cells Inspired by Statistical Mechanics

**DOI:** 10.1038/srep34457

**Published:** 2016-10-06

**Authors:** Christoffer Åberg, Juan A. Varela, Laurence W. Fitzpatrick, Kenneth A. Dawson

**Affiliations:** 1 Centre for BioNano Interactions, School of Chemistry and Chemical Biology, University College Dublin, Belfield, Dublin 4, Ireland

## Abstract

Experimental observations in cell biology have advanced to a stage where theory could play a larger role, much as it has done in the physical sciences. Possibly the lack of a common framework within which experimentalists, computational scientists and theorists could equally contribute has hindered this development, for the worse of both disciplines. Here we demonstrate the usage of tools and concepts from statistical mechanics to describe processes inside living cells based on experimental data, suggesting that future theoretical/computational models may be based on such concepts. To illustrate the ideas, we describe the organisation of subcellular structures within the cell in terms of (density) pair correlation functions, and subsequently use the same concepts to follow nano-sized objects being transported inside the cell. Finally, we quantify an interesting subcellular re-organisation, not previously discerned by molecular biology methods.

Theorists with a foundation in the physical sciences sometimes cross the traditional disciplinary borders to contribute to developments in, for example, very active research arenas such as nanomedicine and nanosafety^1^,^2^, and even physiology^3^,^4^. However, they are often challenged by the absence of a common experimental and theoretical framework within which to contribute, while remaining understood by their own communities. This issue spans generations and sub-disciplines, limiting the ambition with which new levels of computational and experimental infrastructure can be deployed to the key scientific disciplines of today. Such questions have long ago been successfully resolved—indeed in some cases even achieved maturity—in arenas of the physical sciences, such as liquid^5^,^6^ and glass^7^,^8^,^9^ theory. There, the tools to describe experiments and interpret simulations allow for a high degree of interpolation between experimental observations and more phenomenological theories, and are understood by all.

Ultimately, we wish to use the capacity of modern live-cell imaging to build ‘computer models’ of key biological processes inside the cell that are both accurate, capture the important events, and allow a transferable model between experimentalists, computational scientists and theorists. We stress that the issue is not about representing imaging results better, for which there are numerous approaches. Rather, we aim towards using live-cell imaging data to create an analogue computation for the study of living cells. Here we demonstrate the usage of tools and concepts from statistical mechanics to describe processes inside living cells based on such data, suggesting that future theoretical/computational models may be based on such concepts. As a key example, we employ the field of how nano-sized objects (nanoparticles) interact with cells^1^,^2^. We consider this will help build the capacity of scientists to communicate and build substantive theoretical understanding in this, and related, arenas.

While our choice of bionanoscience is purely illustrative, it has some specific merits for our purposes, aside from being important for applications. Broadly speaking, nano-scale objects are recognized and actively internalized by cells (i.e., cells expend their energy), subsequently following intracellular routes originally intended to carry biomolecules for messaging and other processes^1^. Consequently, much effort has been directed towards functionalizing the nanoparticle surface to control the organism and intracellular fate^10^,^11^,^12^. For instance, for genetic medicines intracellular delivery to the nucleus is a key aim. However, in practice, many nanoparticles end up predominantly following the ‘default’ pathway, ultimately accumulating in the lysosomes^1^,^13^,^14^, the degradative compartments of the cell. It is believed that size, shape, surface moieties and—perhaps most important of all—biomolecules adhering to the nanoparticle surface^2^,^15^,^16^ play key roles in determining how cells process nanoparticles, but the nature and drivers of the processes are far from settled. Arguably, this information is crucial if nano-based medicine is to achieve its promises^17^, as well as for the safe implementation of nanotechnology^18^,^19^. Thus, building models describing this particular question—models that can be progressively deepened and understood by many—would itself be of key importance.

In what we will describe here, we consider the statistical mechanical description of glasses^7^,^8^,^9^,^20^ a useful reference-point for the physical theorist. Central issues, while no different from those of a complex liquid in the vicinity of a glass transition, are largely absent from current thinking in the fields we address. Such issues include appropriate levels of description; appropriate and choice of separation of time-scales; definition of the state of the system, and the nature of ‘equilibrium’, steady state, and kinetic (‘ageing’) processes; and how these are to be designated, and computed from experimental data. We use cellular substructures (‘organelles’) that are clearly identified by optical means and sufficiently stationary to meaningfully describe the system over relevant time-scales. Individual cells are fairly self-contained on relatively long times scales, after which cell division (~tens of hours) and exchange of material between cells (~days) take place; organelles move on timescales of ~0.1–1 s within a well-defined intracellular space that can be captured by dynamical microscopy.

We frame much of the description in terms of the time-resolved pair correlation function, *g*_*i*_(*r*;*t*), for different organelles and nanoparticles, together with the corresponding cross pair correlation functions, *g*_*ij*_(*r*;*t*), between these objects^8^. Aside from their ubiquitous appearance in statistical physics^8^, such pair correlations have also been used in the stereology community^21^ to quantify correlations among cells and organelles averaged over cells in tissue samples. Such and similar works have, for example, shown intriguing differences between how cells organise in healthy and cancerous tissue^22^,^23^ or in the healthy brain compared to that of a mouse Alzheimer’s disease model^24^, suggesting the potential for novel discoveries using such tools. Here we use these tools defined and interpreted in living single cells, allowing us to show both the evolution of dynamical processes inside cells, as well as differences between cells.

We start by utilizing pair correlation functions to describe the positions of organelles inside cells, finding a distance in the organelle distribution which appears to be universal in several different human cells. We then demonstrate their use for describing the intracellular transport of nanoparticles, showing the potential for describing dynamical processes. Finally, we illustrate how nanoparticles may impact organelle re-organization in an atypical cell. This is an interesting example, because the nanoparticles used induce no known (i.e., based upon molecular biology methods) biological impact at the average cell population level.

## Results

We used A549 (carcinomic human alveolar basal epithelial) cells and fluorescent 100 nm (nominal diameter) carboxylated polystyrene nanoparticles as a model system, because of the high fluorescence intensity of the nanoparticles and because we have previous experience on the uptake kinetics and intracellular fate of these^1^,^25^, and similar^1^,^25^,^26^, nanoparticles in the same cell line, allowing us to compare our results directly with our previous observations. We may also compare with previous studies on the co-localisation kinetics in related systems^14^,^27^. Supporting experiments were also performed with 1321N1 (astrocytoma human brain glial) and HeLa (adenocarcinomic human cervixal epithelial) cells. Basic physicochemical characterisation of the nanoparticles is reported in Supplementary Table S1. Lysosomes and mitochondria were stained with commercial dyes, and 25–100 three-dimensional images (‘z-stacks’) were acquired in succession using spinning disk confocal microscopy, extending over 100–300 s (depending upon size of cell and dyes used). No significant bleaching nor any signs of phototoxicity were detected under the conditions used for the results reported here.

**Figure 1:**
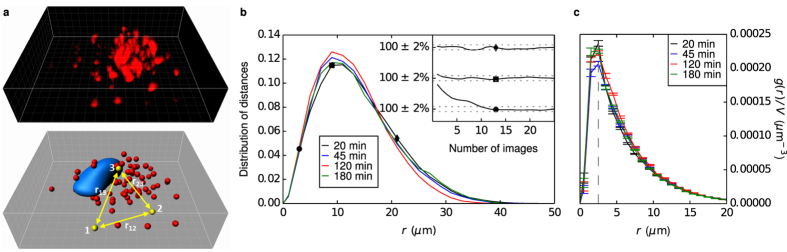
Pair correlation function applied to cells. (**a**) Methododology for calculating the pair correlation function for intracellular organelles. The organelles are fluorescently stained (top) and one can thereby identify individual organelles and their positions (bottom). The distances (*r*_*ij*_) between all organelles are calculated and their distribution determined. Grid spacing: 2 *μ*m (**b**) Lysosome pair correlation function. Different curves correspond to different times after start of the experiment (legend in figure). (inset) Convergence of the pair correlation function with the number of images used to calculate the average. The different curves represent the value of the pair correlation function at a distance of *r* = 3, 9 and 21 *μ*m (indicated with the same symbols in the main graph). Dotted lines shows ±2% of the final average. (**c**) Lysosome-lysosome pair correlation function after normalisation with 4*πr*^2^d*r* (same cell as in panel b). Error bars represent standard error of the mean over 25 images. (Dashed line) Distance of 2.5 *μ*m.

As a proof-of-concept of measuring the pair correlation function between organelles in living single cells, we stained the lysosomes in a cell and acquired 25 three-dimensional images in succession. The positions of the lysosomes were identified by the use of commercial software and the pair correlation function calculated as the distribution of distances between all lysosome pairs (Fig. 1a). We normalised the distributions such that histograms add up to unity, and mostly used it without additional division by 4*πr*^2^dr to better exhibit the full distance dependence.

Figure 1b shows an example of a lysosome pair correlation function determined in this way. The distribution starts at 0, simply because two lysosome cannot overlap so there are no distances between lysosomes smaller than the closest distance of approach (which, if they were all the same size, would be the diameter of a lysosome). The distribution exhibits a clear maximum around 10 *μ*m, subsequently decays towards 0 and vanishes after ~40 *μ*m, corresponding to the size of the cell. The fact that the distribution decays suggests that the lysosomes are not predominantly located at the cell periphery. The pair correlation function therefore gives a geometric description of how (in this case) lysosomes organise within the cell.

We also investigated the robustness and reproducibility of the lysosome pair correlation function. Figure 1b inset shows how the pair correlation function changes with the number of images used in calculating the averages. We may observe that already after roughly 10 images, the average remains within 2% of the final value, suggesting that it would not change appreciably if acquisition was continued. Figure 1b also reports pair correlation functions from measurements on the same cell 25, 100 and 160 min after the first acquisition, showing that the pair correlation function is quite reproducible in time, and certainly the general features are constant in time. All of these remarks are also observed for different cells in the ensemble.

In statistical physics one typically corrects for the distribution of distances growing as 4*πr*^2^ for completely uncorrelated objects (an ideal gas). The underlying assumption of infinite system size is not applicable to cells; nevertheless, at short distances we may disregard this complication and normalise by 4*πr*^2^d*r*. Figure 1c shows the lysosome pair correlation function using this normalisation, to better show features at short distances, exhibiting a clear maximum at around 2.5 *μ*m, which is constant during roughly 3 h. Interestingly, the same peak position is observed for all other cells investigated (see examples in Supplementary Fig. S1), the observation being independent of the dye used to stain the lysosomes (Supplementary Fig. S1). Intriguingly, the same peak position was observed also in two other cell types, 1321N1 (Supplementary Fig. S2) and HeLa (Supplementary Fig. S3) cells, suggesting a universal feature for how lysosomes distribute inside cells. It is worthwhile to note that this distance is not far from the point of nearest approach for lysosomes (diameter is approximately 0.4 *μ*m^28^), suggesting that the statistical ensemble is dominated by near contact of the organelles. Given that lysosomal motions are driven by active cell processes, this is certainly not a packing phenomenon, but instead may be linked to ‘communications’ (for example ‘kiss-and-run’ events^29^) between lysosomes.

In general, the robustness and reproducibility in time of the lysosome pair correlation function (Fig. 1b) shows that the pair correlation function can be defined and measured for lysosomes in single living cells, and other organelles inside the cell can be treated similarly. Furthermore, one observes large changes in the lysosome pair correlation function as a cell divides (Supplementary Fig. S4). These two observations suggest that one may use pair correlation functions to describe the dynamic state of single cells in geometric terms. This is a tempting proposition, for it implies that one may describe both the intracellular transport of nanoparticles, as well as their eventual impact on the cell (not to mention endogeneous processes, such as cell division) within the same framework.

Thus, we continued by extracting a view of the state of single living cells in terms of pair correlation functions for lysosomes, mitochondria and the nucleus. The lysosomes were chosen because they are the end-point of the endo-lysosomal pathway, the typical route of the 100 nm carboxylated polystyrene nanoparticles used in this study^1^,^25^,^26^. The mitochondria, on the other hand, are involved in cell death^30^, and we included them with the hypothesis that cell death would change their spatial organisation in mind. Our experimental setup is limited to two fluorescent dyes, but we could identify also the nucleus (from the absence of fluorescence within it; Supplementary Fig. S5) and included it as an indicator of the general geometry of the cell. The same cell was investigated at different times, each acquisition totaling 25 consecutive three-dimensional images.

**Figure 2:**
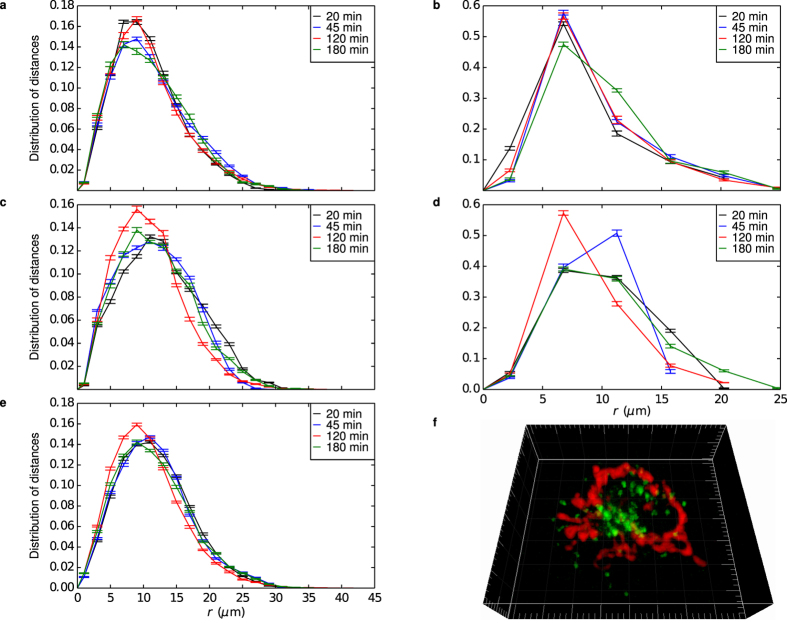
State of a cell described in terms of pair correlation functions. Different curves correspond to different times after start of the experiment (legend in figure) and error bars represent standard error of the mean over 25 images. (**a**) Lysosome-lysosome (**b**) Lysosome-nucleus (**c**) Mitochondrion-mitochondrion (**d**) Mitochondrion-nucleus and (**e**) Lysosome-mitochondrion pair correlation functions. For panels b and d, the fact that there is only one nucleus neccessarily gives poor statistics (the number of samples scales only linearly with the number of lysosomes/mitochondria), and the bin size had to be chosen quite large. (**f**) Three-dimensional rendering showing stained lysosomes (green; note the difference compared to Figs 1a and 3a) and mitochondria (red). Grid spacing 1 *μ*m.

Figure 2 shows the full set of acquired pair correlation functions from the same cell, not normalised by 4*πr*^2^ to better show the full distance behaviour. The lysosome-lysosome pair correlation function exhibits the same general features as observed above (Fig. 1b). Furthermore, we may observe how a large fraction of the lysosomes are in close proximity to the nucleus, as evidenced by a clear maximum at a distance of around 7 *μ*m in the lysosome-nucleus pair correlation function (Fig. 2b). This is likely a reflection of the lysosomes being close to the microtubule organising center^31^. Still, lysosomes can be found in essentially all parts of the cell, as indicated by a substantial pair correlation function also at distances between 10–20 *μ*m. For distances larger than 25 *μ*m the pair correlation function decays to 0, consistent with the size of the cell. All these observations can be corroborated in actual images of the cell (such as Fig. 2f), but the pair correlation function provides a quantitative measure.

The mitochondrion-mitochondrion pair correlation function (Fig. 2c) exhibit the same general features as the lysosome-lysosome pair correlation function (Fig. 2a), though the distribution is somewhat more broad. The mitochondria are also located close to the nucleus (Fig. 2f). This is clearly reflected in the mitochondrion-nucleus pair correlation function (Fig. 2d), which has a maximum around 10 *μ*m. Compared to the lysosomes, the distribution is, however, somewhat more broad, consistent with the same observation for the mitochondrion-mitochondrion pair correlation function (Fig. 2c).

The lysosome-mitochondrion pair correlation function (Fig. 2e) shows a close association of lysosomes and mitochondria, though they are certainly not overlapping. Collectively, the pair correlation functions are all consistent with the view that the lysosomes and mitochondria are mainly located close to the nucleus and close to each other, though not interconnected.

**Figure 3:**
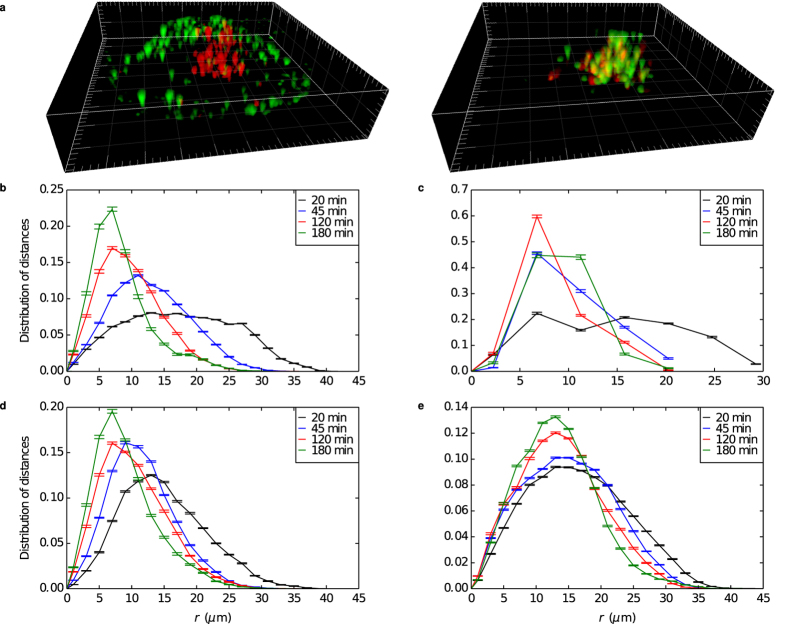
Intracellular transport of nanoparticles in cells. (**a**) Three-dimensional rendering of nanoparticles (green) and lysosomes (red; note the difference compared to Fig. 2f) associated with the cell. Grid spacing 1 *μ*m. (Left) 20 min after exposure, most nanoparticles appears adhered to the outer cell membrane. (Right) 3 h after exposure, nanoparticles can be found inside the cell, several accumulated in lysosomes. The same process can also be seen from the pair correlation functions: (**b**) Nanoparticle-nanoparticle (**c**) nanoparticle-nucleus (**d**) nanoparticle-lysosome and (**e**) nanoparticle-mitochondrion pair correlation function. Different curves correspond to different times after start of the experiment (legend in figure) and error bars represent standard error of the mean over 25 images. Note that, due to the limited number of colours available in our experimental set-up, panel e is from a different cell compared to panels a–d. The results have been adjusted to account for a cluster of nanoparticles being identified as a single object using the procedure outlined in Supplementary Fig. S7. See Supplementary Fig. S8 for the results without this procedure.

Having extracted a description of the dynamic state of single living cells in terms of pair correlation functions, we used the same concept to describe the intracellular transport of nanoparticles. A technical complication is that individual nanoparticles cannot be resolved if closer together than the optical diffraction limit. However, simply ‘counting’ the number of nanoparticles based on their fluorescence can readily remedy this (see Supplementary Information Section Quantification of Nanoparticle Numbers and Supplementary Figs S6 and S7); the major outcomes are in practice anyway independent of this procedure. Cells were exposed to 100 nm carboxylated polystyrene nanoparticles for 10 min, after which the solution was replaced by fresh (nanoparticle-free) medium. At different times after the exposure, 25 consecutive three-dimensional images were acquired, as above. From this exposure around 200–300 nanoparticles (corresponding to 100–200 identified objects) are associated with an A549 cell (of the typical phenotype). Figure 3a shows one example. Shortly after the pulse (Fig. 3a; left), the majority of the nanoparticles are found in the perimeter of the cell, likely adsorbed to the outer side of the plasma membrane, though some may be just being internalized. These observations are consistent with our previous work, based on cell population average data, which showed a significant fraction of nanoparticles adsorbed to the outer cell membrane at such time-scales^25^. However, intriguingly already at these early times one can find a few nanoparticles that are clearly well inside the cell. With time (Fig. 3a; right), nanoparticles can be observed to have moved from the perimeter to what is undoubtedly the inside of the cell, many clearly associated with lysosomes.

Figure 3b–e show a quantitative analysis of the same process in terms of pair correlation functions, again not normalised by 4*πr*^2^ since we are interested in the behaviour at all distances. From the nanoparticle-nucleus pair correlation function (Fig. 3c) it is clear that the nanoparticles move closer to the nucleus with time. The nanoparticle pair correlation function (Fig. 3b) shows how the nanoparticles are initially far apart, with an almost uniform distribution. With time a maximum forms, which progressively moves towards short distances, showing how the nanoparticles move closer together. The nanoparticle-lysosome pair correlation function (Fig. 3d) shows the nanoparticles arriving closer to the lysosomes progressively with time. 2–3 h after the pulse there is a significant number of nanoparticles in lysosomes, as is clear from the fact that the nanoparticle-lysosome pair correlation function does not vanish at the origin. This time-scale is consistent with our previous observations^26^,^27^, as well as those of others^14^. The nanoparticles also move closer to the mitochondria, as revealed by the nanoparticle-mitochondrion pair correlation function (Fig. 3e); still, no co-localisation with mitochondria was observed, as the nanoparticle-mitochondrion pair correlation function is zero at the origin, consistent with previous observations of ours where we have never found these particles in mitochondria^1^,^26^.

Overall, the set of pair correlation functions show a consistent view of the nanoparticles being transported from the cell membrane towards (at least predominantly) the lysosomes. As the lysosomes are chiefly located close to the nucleus (Fig. 2b), where also the mitochondria reside (Fig. 2d), the nanoparticles also arrive close to, but do not co-localise with, the nucleus and the mitochondria. All in all, these conclusions are consistent with our previous works^1^,^26^,^27^. The validity of these conclusions do not depend on the approximate classification (Supplementary Fig. S7) of the number of nanoparticles within an identified fluorescent object, because the same qualitative observations may be drawn from the pair correlation functions not adjusted for the presence of multiple nanoparticles inside an identified object (Supplementary Fig. S8). Geometrical super-resolution could be applied to render a more detailed picture, though without much added value for current discussions. Overall the observations also give clear and meaningful indications of the nanoparticle uptake and intracellular trafficking kinetics, and could be the basis of future more phenomenological theoretical descriptions of those.

Finally, we investigated the eventual impact of the nanoparticles on single living cells within the same framework of pair correlation functions. No evident changes to the lysosome-lysosome or lysosome-nucleus pair correlation functions were observed for the cell shown in Fig. 3a–d (Supplementary Fig. S9). Indeed, this was a general observation for the typical phenotype, as may be expected from the fact that carboxylated polystyrene nanoparticles show no obvious signs of toxicity^1^.

**Figure 4:**
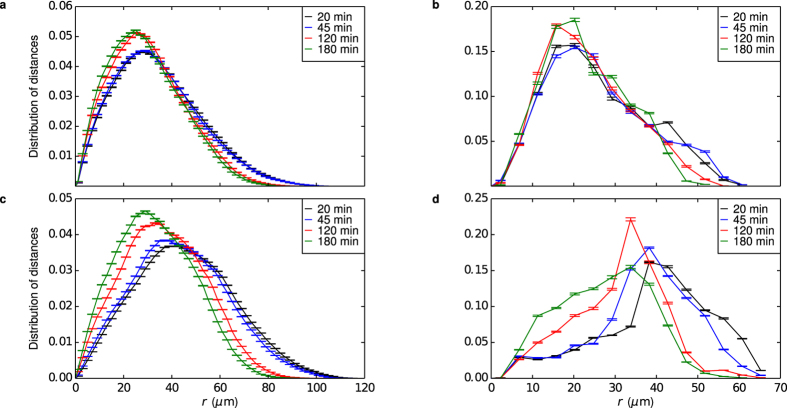
Perturbation to cell described in terms of pair correlation functions, together with the concomitant transport of nanoparticles towards the lysosomes. (**a**) Lysosome-lysosome (**b**) Lysosome-nucleus (**c**) nanoparticle-lysosome and (**d**) nanoparticle-nucleus pair correlation functions. Different curves correspond to different times after start of the experiment (legend in figure) and error bars represent standard error of the mean over 25 images. The results in panel c-d have been adjusted to account for a cluster of nanoparticles being identified as a single object using the procedure outlined in Supplementary Fig. S7; see Supplementary Fig. S10 for the results without this procedure.

However, it is important to stress that not all cells are the same. Hence, it could also be of interest to explore a variety of different cell phenotypes, to identify any novel features there. As an example, we observe a clear trend in the pair correlation functions measured for a phenotypically atypical large cell (Fig. 4). For this cell, the lysosome-lysosome pair correlation function (Fig. 4a) shows the lysosomes moving closer to each other, as nanoparticles enter. Concomitantly, the lysosomes moves closer to the nucleus, as evidenced by the lysosome-nucleus pair correlation function (Fig. 4b). These dynamic changes correlate with the transport of the nanoparticles towards the lysosomes and the nucleus (Fig. 4c,d; for completeness, the same data unadjusted for the optical diffraction limit is shown in Supplementary Fig. S10), suggesting that the nanoparticles are, indeed, the cause. Such phenomena have never previously been reported for nanoparticles, but we may note a parallel in the action of cell starvation^32^. Regardless of the ultimate cause (here possibly pro-autophagic responses), the results clearly demonstrate that a framework of pair correlation functions serves well to describe both the intracellular transport of nanoparticles as well as their eventual geometrical impacts, highlighting the potential for a more ‘precise’ structural science of living systems.

## Discussion

Here we have demonstrated the usage of pair correlation functions as a quantitative measure for describing structure and dynamic processes in single living cells. Intriguingly, we found evidence of universality in how lysosomes distribute inside cells, with a characteristic distance of around 2.5 *μ*m. This distance was observed in all cells investigated, and for several different cell types, covering several different parts of the human body: brain (1321N1), lung (A549) and cervix (HeLa).

Furthermore, the usage of pair correlation functions raises the interesting possibility of describing the state of single living cells within the same framework as dynamical processes, in our case nanoparticle transport. Apart from following the simultaneous evolution of the nanoparticles inside as the cell is undergoing endogenous processes (such as cell division), a more tantalising option is to describe the effect of nanoparticles that impact cells. Thus, while the nanoparticles are being transported within the cell and (potentially) causing damage, the concomitant effects can be described within the same framework. We have exemplified this by showing how the lysosomes in a particular cell re-organized themselves concomitantly with the nanoparticles being taken up and arriving in the lysosomes. This is an interesting example, because the nanoparticles used induce no known (i.e., based upon molecular biology methods) biological impact.

From a broader perspective, we consider that our approach could provide a new and stimulating link between theory, computational science, and cell biology, bridging a gap that has limited the enrichment and possibly key advancements in both arenas. While the multiplicity of times-scales, emergence of limited segments of non-ergodicity, and slow kinetic processes after perturbation are all challenging issues for theory and simulation, they are reminiscent of glassy physics^7^,^8^,^9^, where a quite deep understanding has been gained by the harnessing of such tools—and the intellectual discipline and structure they carry with them. We acknowledge the current unfamiliarity of these issues to both the physical and the biological sciences, and the ambitiousness of such an effort in imaging and computational resources, but suggest that the kinds of ideas presented here could substantially enhance the way physical and biological science engage with each other, to spur future computational/theoretical models of key biological processes inside cells.

## Methods

### Cell culture

Cells were cultured at 37 °C in 5% CO_2_ in Minimum Essential Medium (MEM, with additional L-Glutamine) supplemented with 10% foetal bovine serum (Gibco), 1% penicillin/streptomycin (Invitrogen Corp.) and 1% MEM non-essential amino acids (HyClone). Cells were subcultured 1:3 every second day by incubating them in 0.25% trypsin (Gibco) when they were confluent and resuspending cells in growth medium. Regular mycoplasma tests were carried out, using the mycoAlert kit (Lonza Inc. Allendale, NJ), showing cells to be mycoplasma free.

### Nanoparticles

Yellow-green carboxylated polystyrene nanoparticles (FluoSpheres) with mean diameter of 0.1 *μ*m were purchased from Molecular Probes and used without further chemical modification.

### Spinning disc confocal microscopy

1.3 × 10^5^ cells were seeded onto 35 mm MatTek dishes and incubated for 24 h before carrying out the experiment. Live cells were stained with different organelle dyes in complete MEM at 37 °C and washed before taking the cells to the microscope or adding the nanoparticles. The concentrations and incubation times were: LysoTracker Red and LysoTracker Green (Molecular Probes) at 0.75 *μ*M for 1 h and MitoTracker Red (Molecular Probes) at 0.5 *μ*M for 30 min. Dual color visualization of cell organelles or nanoparticles was performed on a spinning-disk confocal microscopy system consisting of a CSU10 spinning disk unit (Yokogawa Electric corporation) and an iXon EMCCD camera (Andor), mounted on an IX81 inverted microscope (Olympus) with climate control chamber. Nanoparticles and Lysotracker Green were excited with a 488 nm laser line, and Lysotracker Red and MitoTracker Red were excited using a 561 nm laser line. A 60 × 1.35 NA Olympus UPlanSAPO oil immersion objective was used.

### Nanoparticle intracellular transport experiments

Cells were stained with organelle dyes as described above and were subsequently incubated with a concentrated nanoparticle dispersion (100 *μ*g/ml prepared in complete growth medium at 37 °C) for 10 min, after which the medium was removed and samples were washed 5 times with 1 ml PBS at 37 °C. Fresh medium (also at 37 °C) was then added to the cells, and imaging was performed in a live cell chamber, at 37 °C under a 5% CO_2_ and 60% humidity atmosphere. Microscopy was performed as described above.

### Object identification

Object identification was performed using Imaris (Bitplane AG, Zurich). Nanoparticles and lysosomes were identified as ‘spots’ and mitochondria as ‘surfaces’, all based upon the fluorescence of the corresponding dyes.

## Additional Information

**How to cite this article**: Åberg, C. *et al*. Spatial and Structural Metrics for Living Cells Inspired by Statistical Mechanics. *Sci. Rep.* **6**, 34457; doi: 10.1038/srep34457 (2016).

## Supplementary Material

Supplementary Information
