# Supplementary material for: Spatial and Structural Metrics for Living Cells Inspired by Statistical Mechanics
Source: Sci Rep. 2016 Oct 6;6:34457. doi: 10.1038/srep34457 (PMC5052623; doi:10.1038/srep34457)
Supplement: Supplementary Information [file srep34457-s1.pdf]

Supplementary Information
Spatial and Structural Metrics for Living Cells Inspired by Statistical Mechanics

Christoffer Aberg, Juan A. Varela, Laurence W. Fitzpatrick, and Kenneth A. Dawson

Centre for BioNano Interactions, School of Chemistry and Chemical Biology, University College Dublin, Belfield, Dublin 4, Ireland

Supporting Methods

Nanoparticles

Yellow-green carboxylated polystyrene nanoparticles (FluoSpheres) with mean diameter of $0.1\,\mu$m were purchased from Molecular Probes and used without further chemical modification. Size and ζ-potential were determined using a Malvern Zetasizer Nano Series, diluting the nanoparticles to $50\,\mu$g/ml in water and Phosphate Buffered Saline (PBS), respectively, before measurement. Measurements were conducted at pH 7.0 and 25 °C.

Nanoparticle dispersion characterisation

Visualisation of nanoparticles in dispersion was performed on a spinning disk confocal microscopy system (see Methods section in main text). To perform single particle tracking analysis, nanoparticles were diluted both in 100% glycerol at 40 °C and water at 25 °C. Two dimensional images were acquired at sampling rates ranging from 50 to 100 frames per second, while three-dimensional images were acquired at roughly 2 frames per second.

Quantification of Nanoparticle Numbers

The nanoparticles used in this work are smaller than the optical diffraction limit, implying that individual nanoparticles cannot be resolved if close enough together. This complication is important to consider as nanoparticles may be taken up together, cluster intracellularly *en route* or concentrate in their final organelle. Indeed, we indirectly observed this occuring in our system. Thus, the nanoparticle fluorescence distribution in dispersions where the nanoparticles remain non-agglomerated exhibits a single, albeit broad, peak (Supplementary Fig. S6). In contrast, inside cells the fluorescence distribution is markedly broader, and growing broader with time (Supplementary Fig. S7). Concomitant with this shift in fluorescence intensity, the number of identified objects inside the cell also decreases (Supplementary Fig. S7). Overall, the observations are consistent with the nanoparticles clustering too close to be individually resolved, hence reducing the number of objects that can be identified while increasing their fluorescence, as has been described previously[1]. Unfortunately, the fluorescence distribution of single nanoparticles is too broad (Supplementary Fig. S6) to unequivocally classify the number of nanoparticles in an identified fluorescent object. As a reasonable first approximation, we divide the intensity distribution into equidistant intervals corresponding to, respectively, single nanoparticles, dimers, trimers etc. (Supplementary Fig. S7). Though rough, this procedure drastically reduces the variation in the number of detected nanoparticles (Supplementary Fig. S7). The shortcomings introduced by the optical diffraction limit could potentially be remedied in future studies by achieving a more narrow fluorescence distribution of the nanoparticles, or by using emerging super-resolution techniques, such as stimulated emission depletion (STED) microscopy[2].

In order to demonstrate that the nanoparticle fluorescence distribution observed inside cells (Supplementary Fig. S7a-b) can be analysed based on a rather broad fluorescence distribution of single nanoparticles, we had to proceed in several steps:

We started by measuring the fluorescence distribution of the nanoparticles in disperson, rather than inside cells, because there we can simultaneously monitor if the nanoparticles are, indeed, single objects. We thus measured the fluorescence using microscopy and under similar settings as used for the cell experiments. However, for the cell experiments data was acquired in three dimensions, which limits the speed with which data can be acquired. In order to identify the nanoparticles in three dimensions also in solution, we thus had to disperse them in a dispersant in which they move sufficiently slowly. Since the nanoparticles diffuse too rapidly in aqueous solutions, we instead dispersed them in glycerol.

The results (Supplementary Fig. S6a) show a distribution with a main peak together with a small shoulder at higher intensities. The main peak likely represent single nanoparticles, but is rather broad, potentially either from a heterogeneous

Table S1. Basic physicochemical characterisation of nanoparticle dispersions.

	Diameter (nm)[a]	PDI[b]	ζ-potential (mV)[c]
Water, 25 °C	114 ± 1	0.04	-
PBS, 25 °C	114 ± 4	0.01	-34 ± 2
cMEM, 37 °C[d]	117 ± 2	0.25	-11 ± 1

[a] z-average hydrodynamic diameter extracted by cumulant analysis of dynamic light scattering data.
[b] Polydispersity index from cumulant fitting of dynamic light scattering data.
[c] Measured using laser doppler velocimetry.
[d] Data reproduced from ref. 5.

loading of the fluorescent dye in the (commercial) nanoparticles and/or from fluctuations in intensity. The shoulder to the right of the main peak we argue is due to nanoparticle-pairs, too close together to be resolved individually in the microscope.

In order to demonstrate that the main peak indeed corresponds to single nanoparticles, we tracked the motion of the objects and calculated their mean square displacement (Supplementary Fig. S6b). The resulting diffusion coefficient is consistent with a diameter of 106 nm, using the Stokes-Einstein equation and literature values for the viscosity of glycerol[3]. This is in good agreement with the nominal size, suggesting that the identified objects are, indeed, individual nanoparticles. (The presence of a few nanoparticle pairs, as in the shoulder to the right of the main peak, does not influence the mean square displacement analysis significantly.)

As a validation of the methodology for measuring the diffusion coefficient of nanoparticles using microscopy we performed similar experiments in water, because there we can readily compare with results from dynamic light scattering. Since this was just a validation of the methodology, we could acquire data in two-dimensions to overcome the limit of slow acquisition. The mean square displacement (Supplementary Fig. S6c) is consistent with a diameter of 114 nm, again using the Stokes-Einstein equation and literature values for the viscosity of water[4]. This is in excellent agreement with the diameter measured using dynamic light scattering (Supplementary Table S1), showing that the methodology is sound.

We may also note that the fluorescence intensity distribution of the nanoparticles in water (Supplementary Fig. S6d) is also rather broad, again consistent with a rather heterogeneous intensity of single nanoparticles.

References

1. Wang, Z., Tiruppathi, C., Minshall, R. D. & Malik, A. B. Size and dynamics of caveolae studied using nanoparticles in living endothelial cells. *ACS Nano* **3**, 4110–4116 (2009).

2. Schübbe, S. *et al.* Size-dependent localization and quantitative evaluation of the intracellular migration of silica nanoparticles in Caco-2 cells. *Chem. Mater.* **24**, 914–923 (2012).

3. Segur, J. B. & Oberstar, H. E. Viscosity of glycerol and its aqueous solutions. *Ind. Eng. Chem.* **43**, 2117–2120 (1951).

4. Lide, D. R. & Haynes, W. M. (eds.) *CRC Handbook of Chemistry and Physics* (CRC Press, Boca Raton, Florida, 2009), 90 edn.

5. Kim, J. A., Åberg, C., Salvati, A. & Dawson, K. A. Role of cell cycle on the cellular uptake and dilution of nanoparticles in a cell population. *Nat. Nanotechnol.* **7**, 62–68 (2012).

6. Lesniak, A. *et al.* Nanoparticle adhesion to the cell membrane and its effect on nanoparticle uptake efficiency. *J. Am. Chem. Soc.* **135**, 1438–1444 (2013).

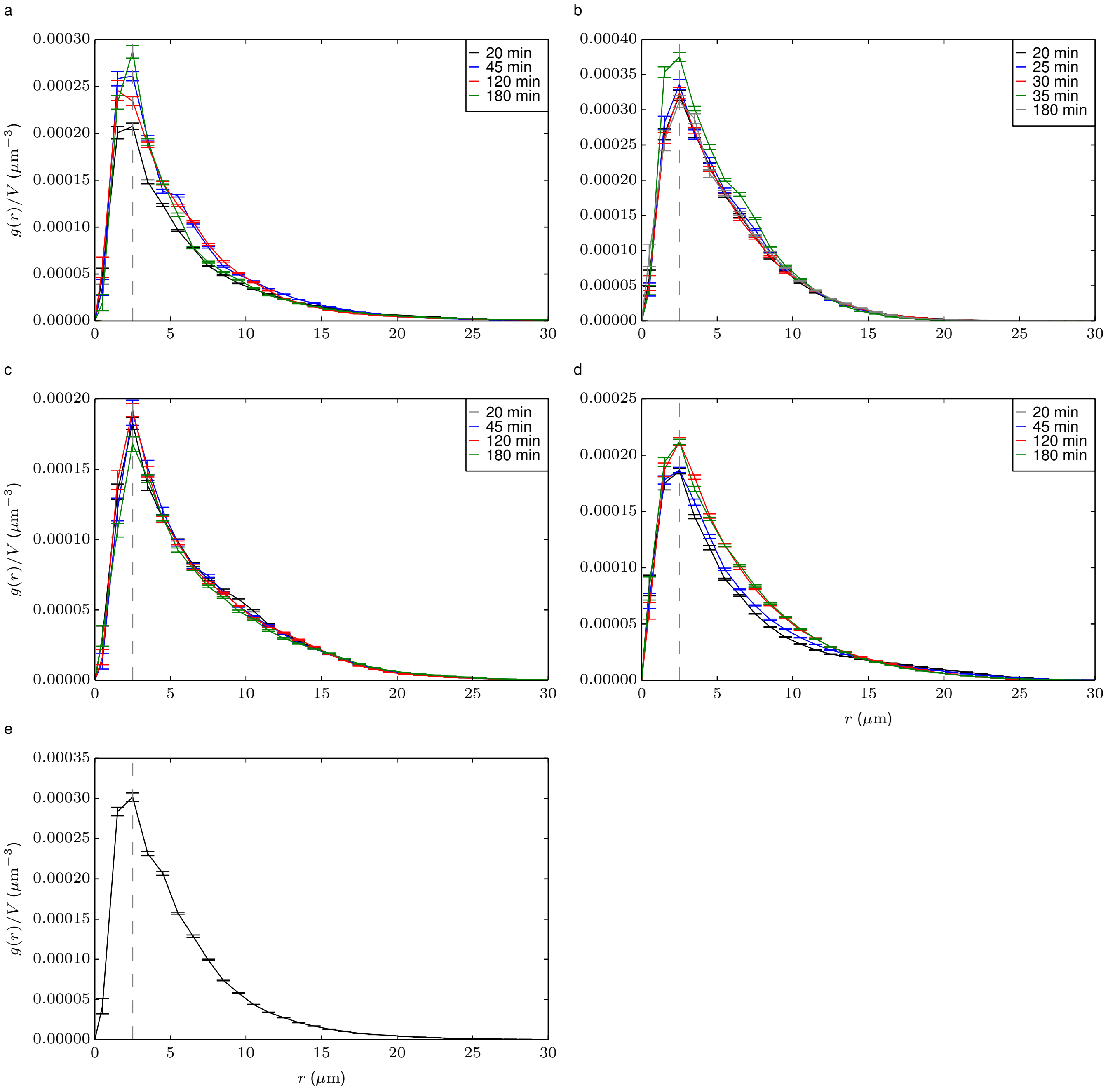

Figure S1. **Lysosome-lysosome pair correlation functions for several different A549 cells.** For the experiments shown in panels a-b and e, LysoTracker Red was used; for panels c-d, LysoTracker Green (together with MitoTracker Red for the mitochondria; not shown here). Different curves correspond to different times after start of the experiment (legend in figure; for panel e, no time-course was investigated) and error bars represent standard error of the mean over 25 images. (Dashed line) Distance of 2.5 μm.

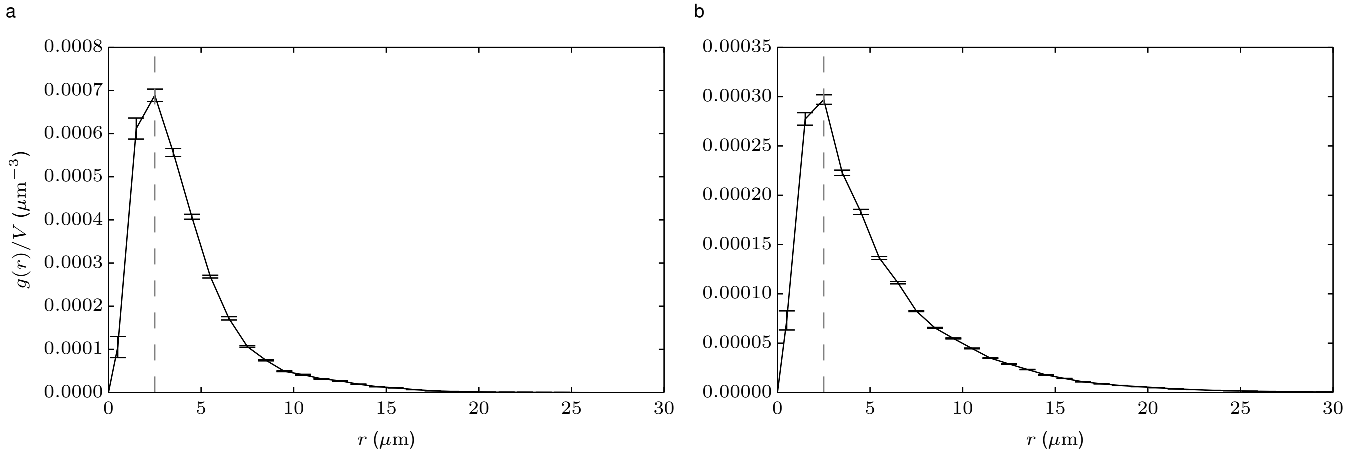

Figure S2. **Lysosome-lysosome pair correlation functions for two 1321N1 cells.** LysoTracker Red was used in both cases. Error bars represent standard error of the mean over 100 images. (Dashed line) Distance of 2.5 μm.

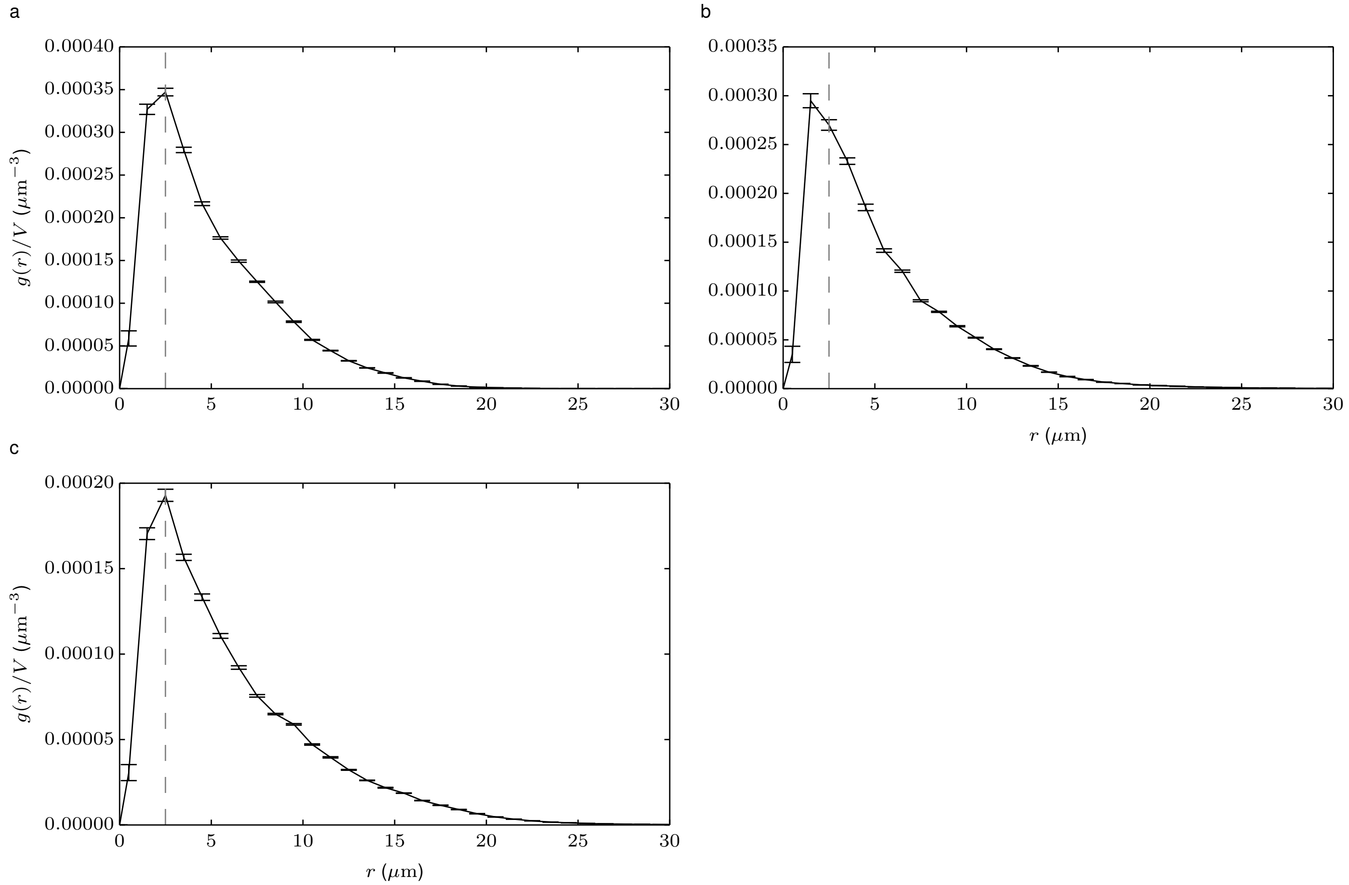

Figure S3. **Lysosome-lysosome pair correlation functions for three HeLa cells.** LysoTracker Red was used in all cases. Error bars represent standard error of the mean over 25 images. (Dashed line) Distance of 2.5 μm.

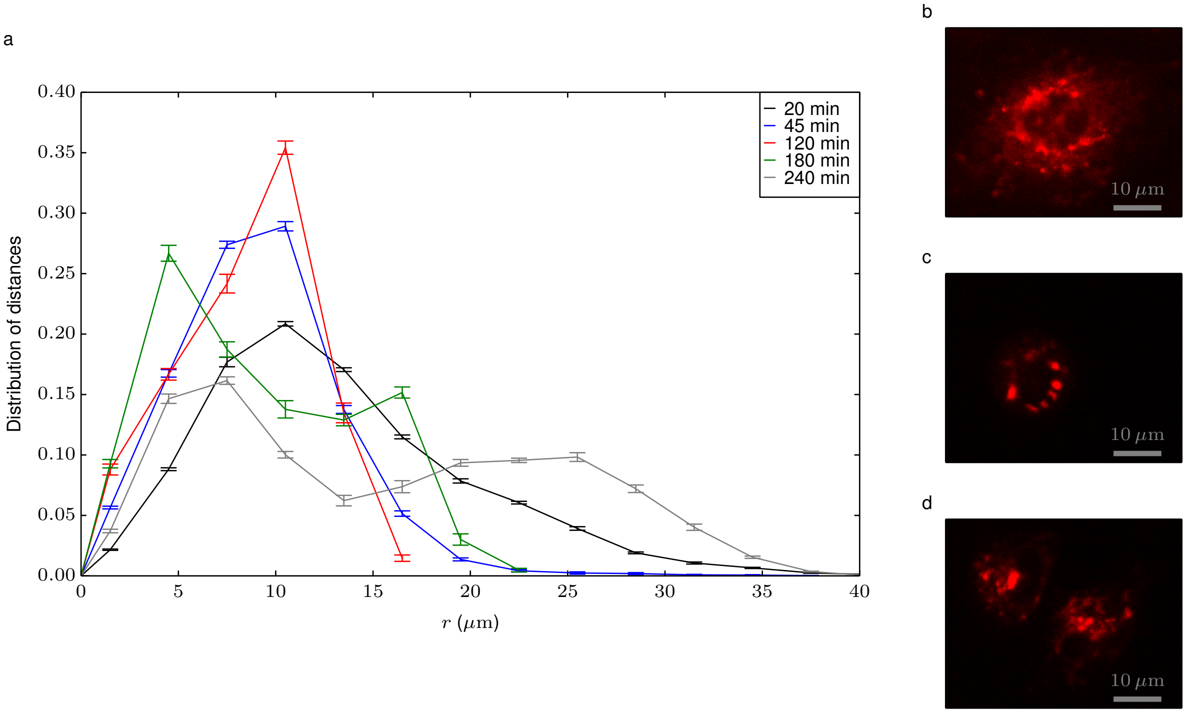

Figure S4. Dividing cell. a, Lysosome pair correlation function. At the first time-point investigated (20 min) the distribution looks similar to other cells [*cf.* Fig. 1b and 2a]. With time the lysosomes gather closer together (45 and 120 min). After 180 min there are two nuclei present, which is also reflected in the distribution which shows two local maxima. Finally, after 240 min two daughter cells are present. Error bars represent standard error of the mean over 25 images. **b-d,** The same process as seen directly from the lysosomal fluorescence. Note that compared to Fig. 2f lysosomes are here in red. One confocal plane averaged over 25 frames, 20, 120 and 240 min after the start of the experiment.

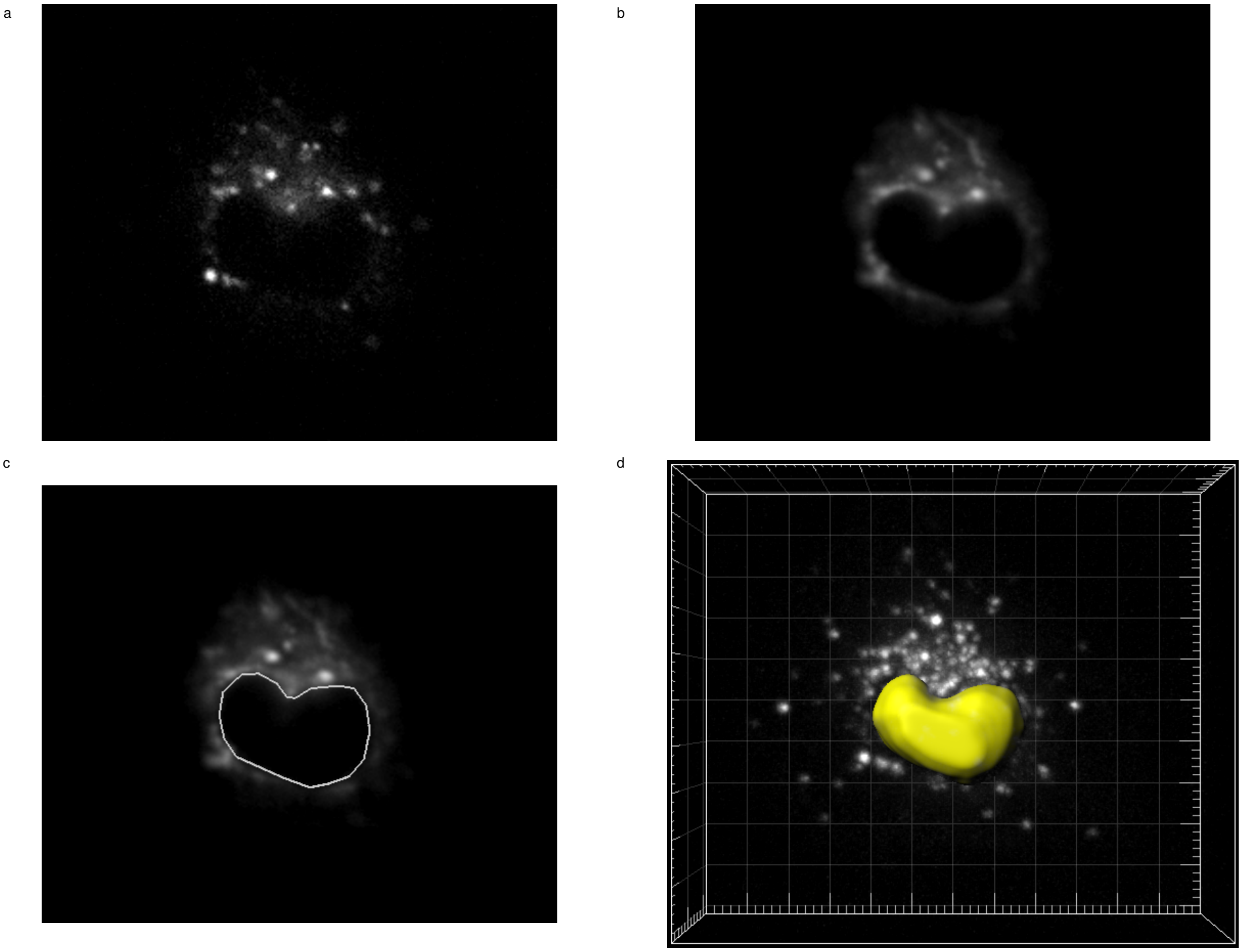

Figure S5. Identification of nucleus without specific staining, using (the absence of) LysoTracker fluorescence. a, LysoTracker fluorescence from a single confocal plane of a cell, showing individual LysoTracker-stained objects. **b,** When the LysoTracker fluorescence from the same confocal plane is averaged over 25 consecutive frames, the outline of the nucleus appears clearly. **c,** From this outline an approximate identification of the nucleus in each confocal plane (where the nucleus is present) is performed. **d,** Based on the outline in each confocal plane, a full three-dimensional reconstruction of the nucleus can be found. Here the reconstruction is shown together with the maximum intensity projection of LysoTracker fluorescence from the full cell.

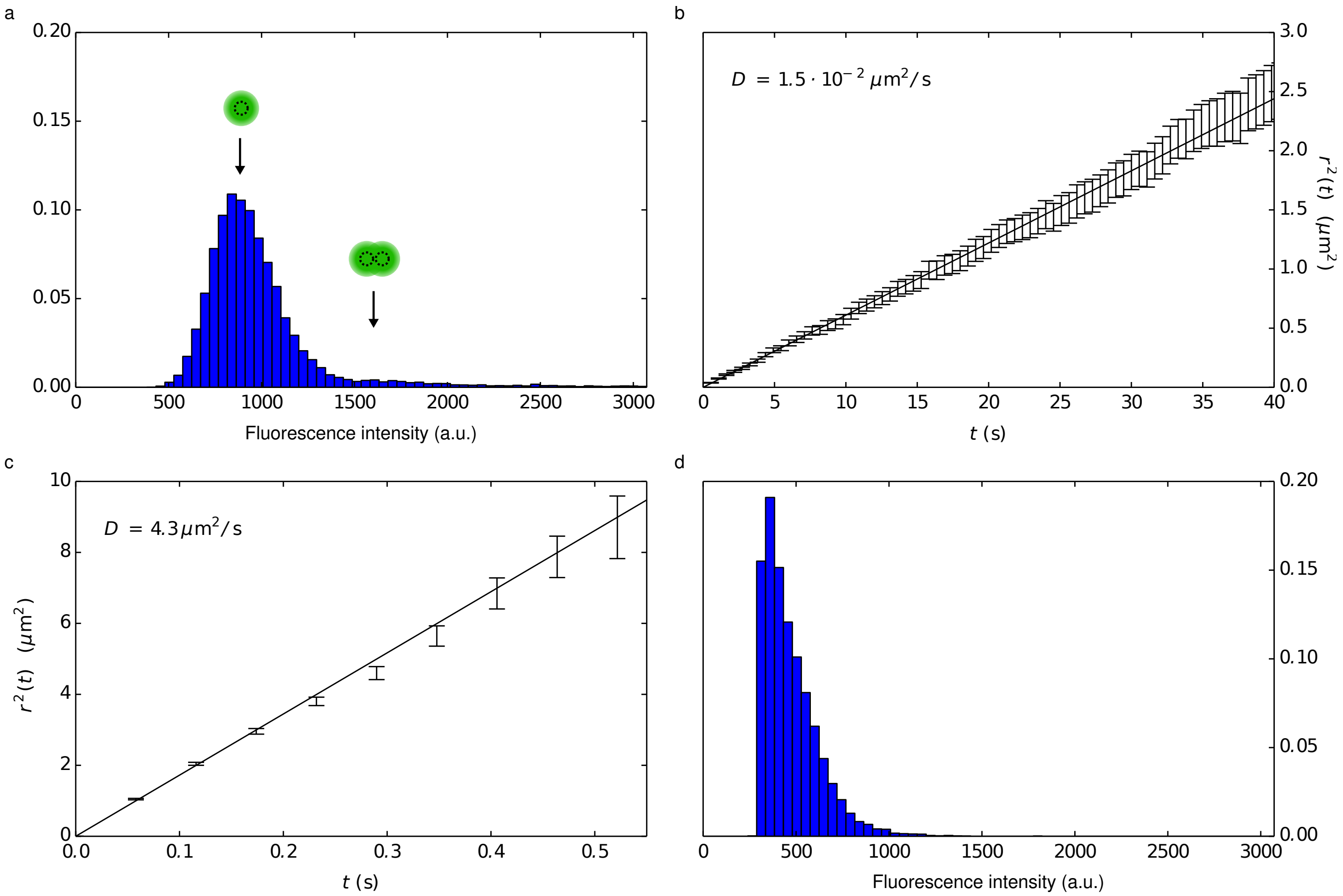

Figure S6. Fluorescence intensity distribution of individual nanoparticles as measured by microscopy. a, Fluorescence intensity distribution of nanoparticles dispersed in glycerol during acquisition of three-dimensional images, similarly to the cell experiments. **b,** Mean square displacement of the same objects. Only the motion in the *xy*-plane was characterised, due to the limited resolution in the *z* direction. Error bars represent standard error of the mean. A fit to the data (solid line) gives an estimated diameter of 106 nm from the Stokes-Einstein equation and literature values for the viscosity of glycerol[3]. **c,** Mean square displacement of identified nanoparticle-objects dispersed in water. The diffusion coefficient estimated from the fit (solid line) corresponds to a diameter of 114 nm, again using the Stokes-Einstein equation and literature values for the viscosity of water[4]. **d,** Fluorescence intensity distribution of the same objects as in panel c.

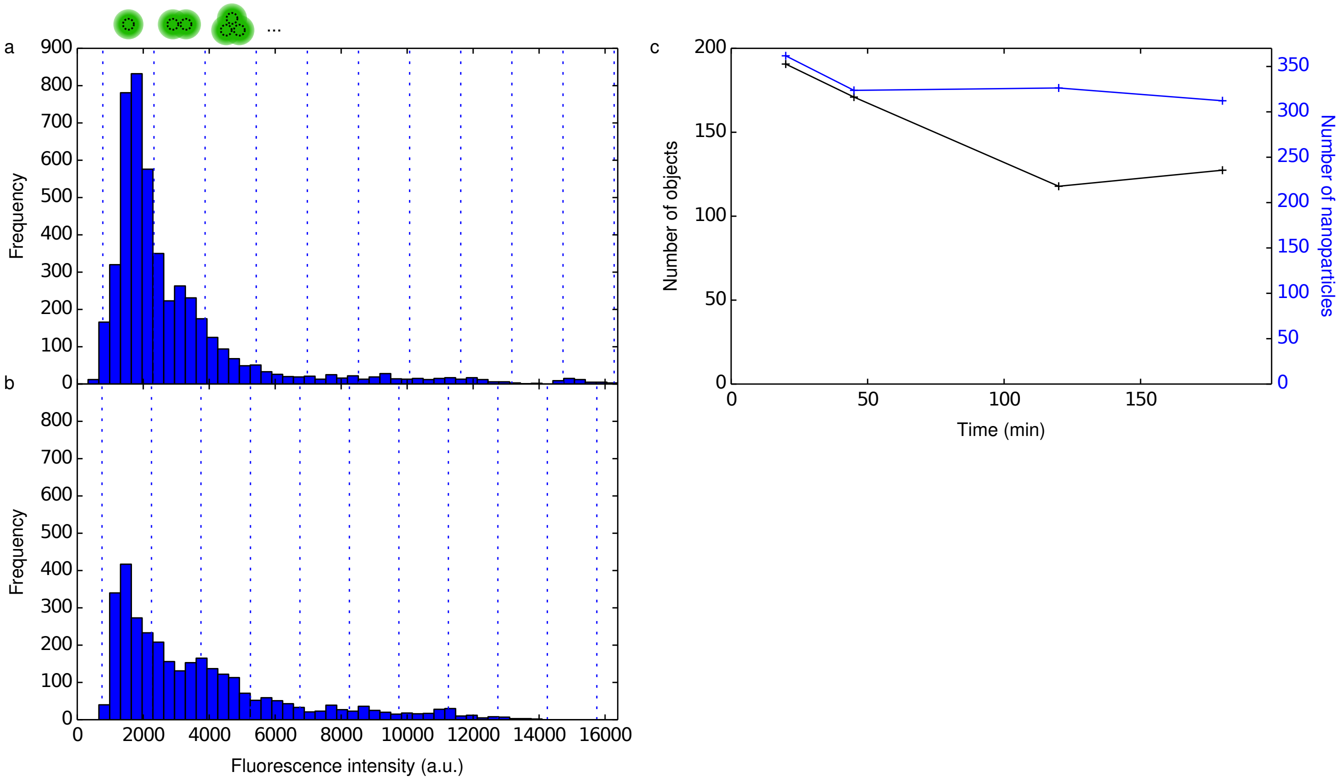

Figure S7. Approximate enumeration of nanoparticles, as opposed to identified fluorescent objects. a-b, Distribution of fluorescence intensity of identified objects 20 min and 3 h after nanoparticle exposure. With time the distribution clearly shifts towards higher intensities. Since the nanoparticles dispersed in solution exhibit (essentially) a single peak in intensity (Supplementary Fig. S6; note, however, that the absolute intensities cannot be compared), the broad distributions are most likely a reflection of several nanoparticles too close together to be resolved individually in the microscope. Assuming, for simplicity, that the fluorescence of multiple nanoparticles in close proximity is proportional to the number of nanoparticles, we can set boundaries on which parts of the distributions correspond to single nanoparticles, dimers, trimers etc. (dotted lines). **c,** Number of identified objects as a function of time. Before considering multiple nanoparticles being identified as a single object, the number of nanoparticles decreases significantly with time (black; left axis). However, with the rough identification of the number of nanoparticles in a fluorescence object (panel a-b) the number of nanoparticles remains remarkably constant with time (blue; right axis). The decrease between 20 and 45 min could be due to some nanoparticles that have not yet been internalised deattaching from the outer cell membrane into solution (a process that has been described previously[6]).

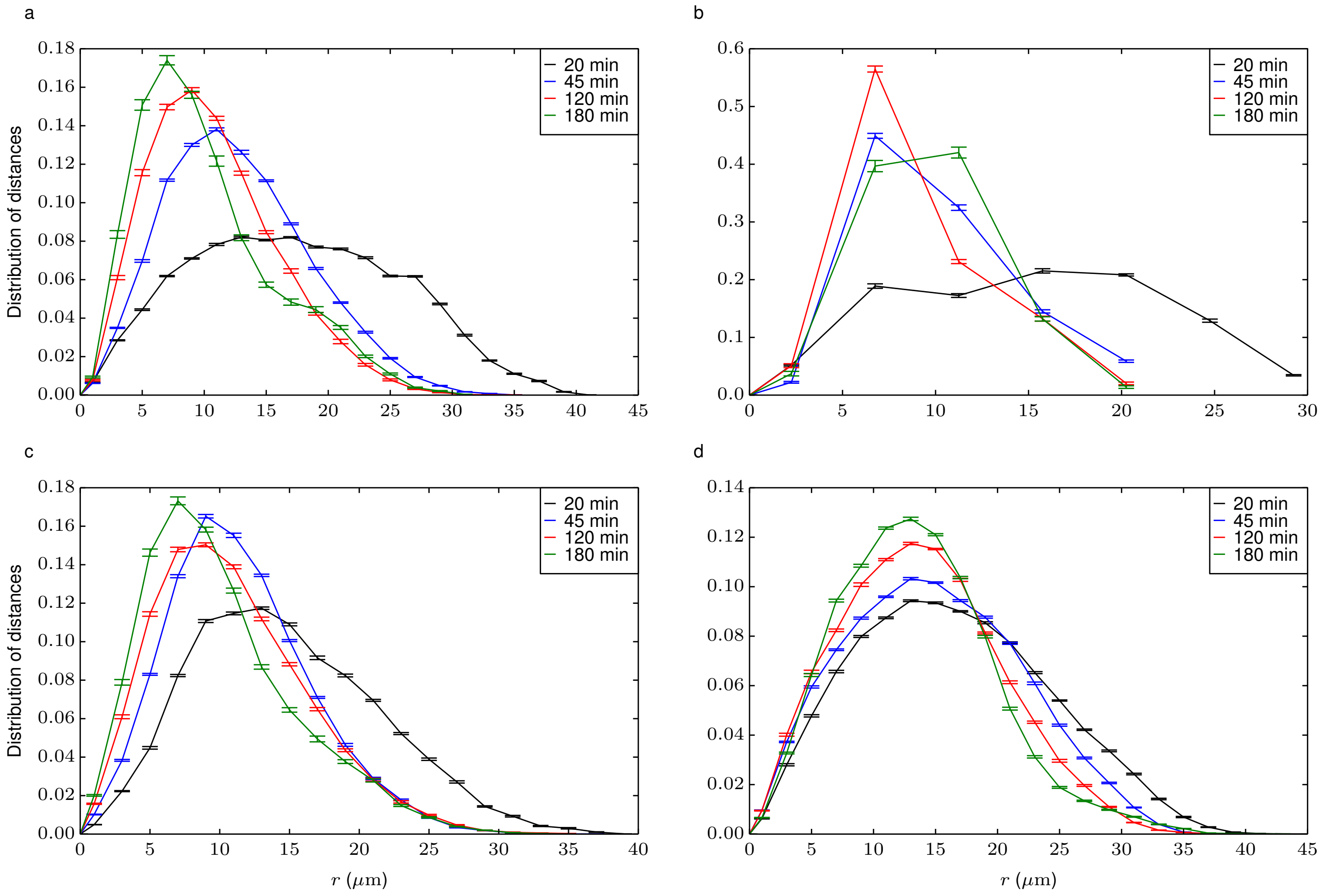

Figure S8. Intracellular transport of nanoparticles in cells in terms of pair correlation functions. The same data as shown in Fig. 3, but not adjusted to account for individual nanoparticles not being well-resolved. **a,** Nanoparticle-nanoparticle **b,** nanoparticle-nucleus **c,** nanoparticle-lysosome and **d,** nanoparticle-mitochondrion pair correlation function. Different curves correspond to different times after start of the experiment (legend in figure) and error bars represent standard error of the mean over 25 images. Note that, due to the limited number of colours available in our experimental set-up, panel d is from a different cell compared to panels a-c. A three-dimensional rendering of the process shown in panels a-c can be seen in Fig. 3a.

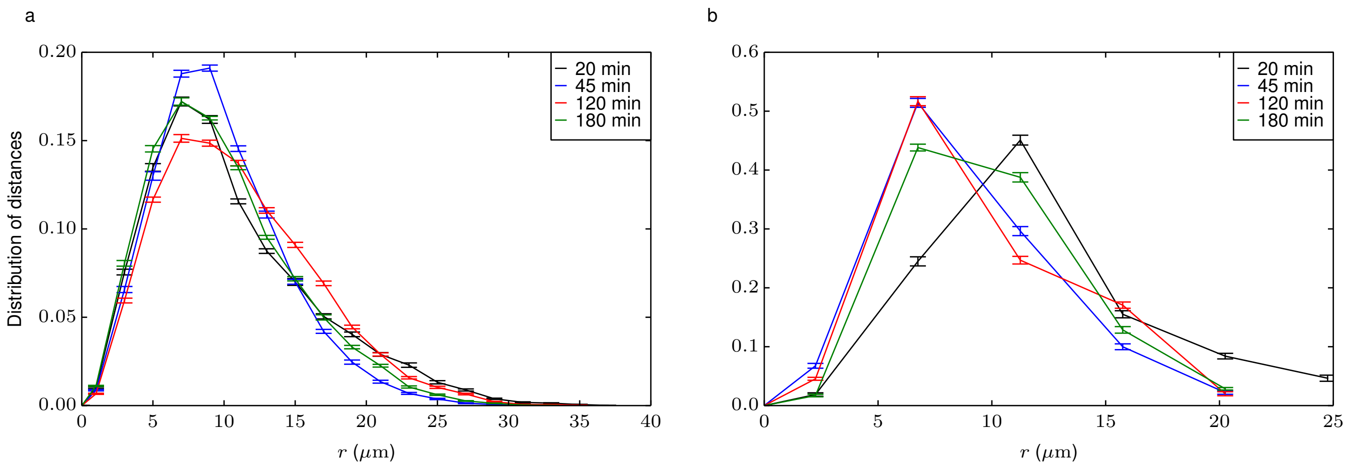

Figure S9. Potential perturbation to a cell due to nanoparticle uptake described in terms of pair correlation functions. Data from the same cell as shown in Fig. 3a-d. **a,** Lysosome-lysosome and **b,** Lysosome-nucleus pair correlation functions. Different curves correspond to different times after start of the experiment (legend in figure) and error bars represent standard error of the mean over 25 images. No clear trend can be seen, suggesting no (with pair correlation functions) discernable impact on the cell.

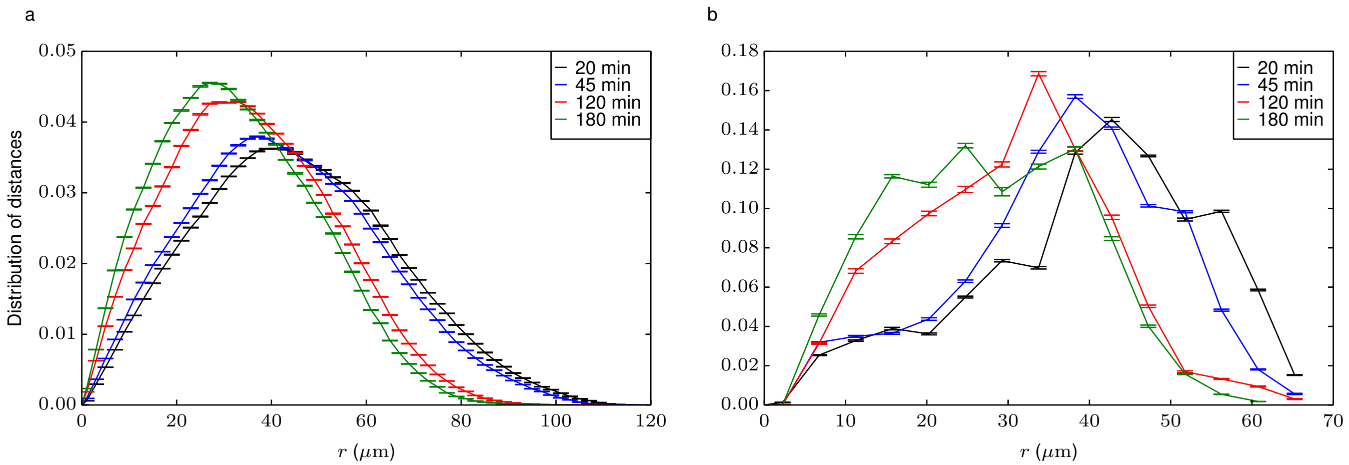

Figure S10. Intracellular transport of nanoparticles in a cell in terms of pair correlation functions. The same data as shown in Fig. 4d-e, but not adjusted to account for individual nanoparticles not being well-resolved. **a,** Nanoparticle-lysosome and **b**, Nanoparticle-nucleus pair correlation functions. Different curves correspond to different times after start of the experiment (legend in figure) and error bars represent standard error of the mean over 25 images.
